# Exosomal MicroRNA and Protein Profiles of Hepatitis B Virus-Related Hepatocellular Carcinoma Cells

**DOI:** 10.3390/ijms241713098

**Published:** 2023-08-23

**Authors:** Valentina K. Todorova, Stephanie D. Byrum, Samuel G. Mackintosh, Azemat Jamshidi-Parsian, Allen J. Gies, Charity L. Washam, Samir V. Jenkins, Timothy Spiva, Emily Bowman, Nathan S. Reyna, Robert J. Griffin, Issam Makhoul

**Affiliations:** 1 Department of Internal Medicine/Division of Hematology/Oncology, University of Arkansas for Medical Sciences, Little Rock, AR 72205, USA; makhoulissam@uams.edu; 2 Department of Biochemistry and Molecular Biology, University of Arkansas for Medical Sciences, Little Rock, AR 72205, USA; sbyrum@uams.edu (S.D.B.); mackintoshsamuelg@uams.edu (S.G.M.); giesallenj@uams.edu (A.J.G.); cwasham@uams.edu (C.L.W.); 3 Department of Radiation Oncology, University of Arkansas for Medical Sciences, Little Rock, AR 72205, USA; jamshidiazema@uams.edu (A.J.-P.); svjenkins@uams.edu (S.V.J.); rjgriffin@uams.edu (R.J.G.); 4 Biology Department, Ouachita Baptist University, Arkadelphia, AR 71998, USA; taspiva@uams.edu (T.S.); emedmondson@uams.edu (E.B.); reynan@obu.edu (N.S.R.)

**Keywords:** microRNA sequencing, mass spectrometry, proteomics, high-grade HCC cells, secretions, extracellular vesicle

## Abstract

Infection with hepatitis B virus (HBV) is a main risk factor for hepatocellular carcinoma (HCC). Extracellular vesicles, such as exosomes, play an important role in tumor development and metastasis, including regulation of HBV-related HCC. In this study, we have characterized exosome microRNA and proteins released in vitro from hepatitis B virus (HBV)-related HCC cell lines SNU-423 and SNU-182 and immortalized normal hepatocyte cell lines (THLE2 and THLE3) using microRNA sequencing and mass spectrometry. Bioinformatics, including functional enrichment and network analysis, combined with survival analysis using data related to HCC in The Cancer Genome Atlas (TCGA) database, were applied to examine the prognostic significance of the results. More than 40 microRNAs and 200 proteins were significantly dysregulated (*p* < 0.05) in the exosomes released from HCC cells in comparison with the normal liver cells. The functional analysis of the differentially expressed exosomal miRNAs (i.e., mir-483, mir-133a, mir-34a, mir-155, mir-183, mir-182), their predicted targets, and exosomal differentially expressed proteins (i.e., POSTN, STAM, EXOC8, SNX9, COL1A2, IDH1, FN1) showed correlation with pathways associated with HBV, virus activity and invasion, exosome formation and adhesion, and exogenous protein binding. The results from this study may help in our understanding of the role of HBV infection in the development of HCC and in the development of new targets for treatment or non-invasive predictive biomarkers of HCC.

## 1. Introduction

Hepatocellular carcinoma (HCC) is the most common type of primary liver cancer and one of the main causes of cancer death worldwide [1,2]. The 2-year survival rate in the United States is less than 50% and the 5-year survival rate is only 10% [3]. The incidence of HCC in the United States is relatively low but is the most rapidly growing cause of cancer-related death in men, projected to become the third-leading cause of cancer-related death by 2030 [4]. Despite the extensive research and widely reported molecular classification of HCC, there has been no significant progress on the targeted therapies for HCC patients [5]. Major risk factors for HCC include hepatitis C virus (HCV) or hepatitis B virus (HBV) infection, alcoholic cirrhosis, and nonalcoholic fatty liver disease. Integration of HBV fragments into the human genome happens in more than half of HBV-infected HCC [6]. HBV is an enveloped, partially double-stranded DNA virus, which replicates through reverse transcription of an RNA intermediate [7]. HBV replication occurs within the viral capsid, containing viral structural proteins in the cytosol of the hepatocytes. The partially double-stranded genome of the virus is delivered to the hepatocyte nucleus, where the genome is repaired by host cell DNA repair machinery to generate covalently closed circular DNA (cccDNA), which forms a mini chromosome serving as a template for all HBV RNA transcripts [8]. Pregenomic RNA (pgRNA), enclosed in capsids, is covalently linked to the viral DNA polymerase/reverse transcriptase (RT), which reverse transcribes the first DNA strand of the HBV genome by using pgRNA as the template. The second DNA strand is synthesized to various lengths, giving rise to the partially double-stranded genome of HBVseeger [9,10]. Four kinds of HBV antigens have been described, including HBxAg, HBsAg, HBcAg, and HBeAg [11].

Exosomes are the major component of extracellular vesicles (EVs) (30–150 nm), which recently have been implicated in cancer progression and metastasis by enhancing angiogenesis, inducing drug resistance, modulating anti-tumor immune mechanisms, and promoting metastatic niche formation [12,13,14,15]. EVs, including exosomes, contain various molecular constituents of their cell of origin, including miRNA, mRNA, proteins, and lipids, which may potentially be used as biomarkers [16,17,18]. Exosomes transfer their contents to the target cells via interactions with their receptors on the recipient cells with which they fuse [19,20]. Fusion of EVs with target cells can transfer receptors on the EV surface to the recipient cell plasma membrane [21], as well as RNA, DNA, lipids, and proteins [22,23]. Many viruses utilize exosomes as another route of transmission, with the additional benefit of being at least partially shielded from immune responses [24]. EVs can influence the surrounding microenvironment by contributing to its remodeling and degradation, including in cancer establishment and progression [25,26,27]. For example, tumor-derived EVs promote angiogenesis by activating angiogenic signaling pathways in endothelial cells (EC) [28,29]. In our previous study, we have demonstrated that HCC cells Hepg2 induced differentiation and angiogenic activity of ECs through a release in the culture media of exosomes with elevated expression of ephrin-B2 and Delta-like 4 ligand (DLL4), known to initiate arterial phenotype [30]. Several studies have shown that exosomes can play an important role in a variety of viral and bacterial infections, including viral hepatitis [31,32,33], by regulating pathogen uptake and replication, as well as by inhibition of the host immune response [34,35].

In this study, we have characterized exosome proteins and microRNA released in vitro from HBV-associated human HCC cell lines and immortalized normal hepatocyte cell lines using mass spectrometry and microRNA sequencing.

## 2. Results

### 2.1. Isolation and Identification of Exosomes

**Figure 1:**
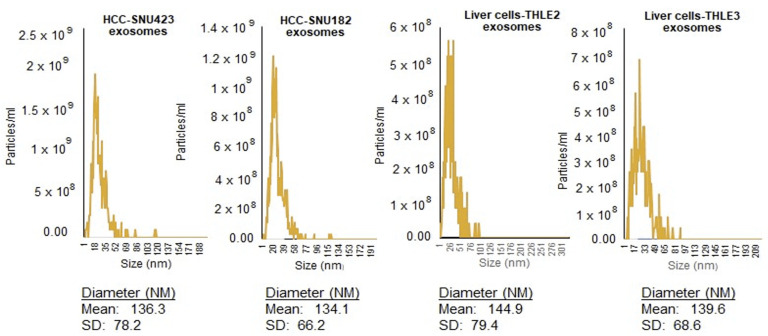
Nanoparticle analysis (NTA) of exosomes released from HBV-related HCC cell lines SNU423 and SNU182, and normal liver cells THLE2 and THLE3. Data represent concentration and size distribution of exosomes released purified from culture media, collected 48 h after cell starvation.

Exosomes were extracted from cell culture media using ultracentrifugation and by ExoQuick-TC-ULTRA. Nanoparticle tracking analysis of the exosomes released from HVB-related HCC cell lines SNU423, SNU182, and immortalized hepatic cell lines THLE2 and THLE3 (controls), was used to determine exosomes’ diameters, which ranged between 30 and 200 nm (Figure 1).

The immortalized human hepatic cell lines THLE2 and THLE3 are often used as a model of normal hepatocytes instead of liver biopsies [36]. THLE2 and THLE3 cell lines were established from normal human liver epithelial cells obtained from two different non-diseased donors with a recombinant simian virus 40 T antigen [37]. THLE2 and THLE3 are nontumorigenic when injected into athymic nude mice, have near-diploid karyotypes, and do not express alpha-fetoprotein. These cells express cytokeratin 18 and albumin in their early passage, and several enzymes involved in metabolism of chemical carcinogens (i.e., epoxide hydrolase, NADPH cytochrome P450 reductase, superoxide dismutase, catalase, glutathione S-transferases, and glutathione peroxidase) [38]. Immortalized hepatic cell lines are widely used for research instead of liver biopsies due to their unlimited growth and stable phenotype, streamline standardized culture protocols and assay reproducibility, and lower cost [36,39]. Primary human cells, isolated from liver tissue as hepatocytes or non-parenchymal cells, better resemble the in vivo phenotype [36,40,41]. However, their limited culture time and availability, especially for non-pathological (“healthy”) controls, as well as sample heterogeneity, are disadvantages [42,43].

### 2.2. Exosomal microRNA

**Figure 2:**
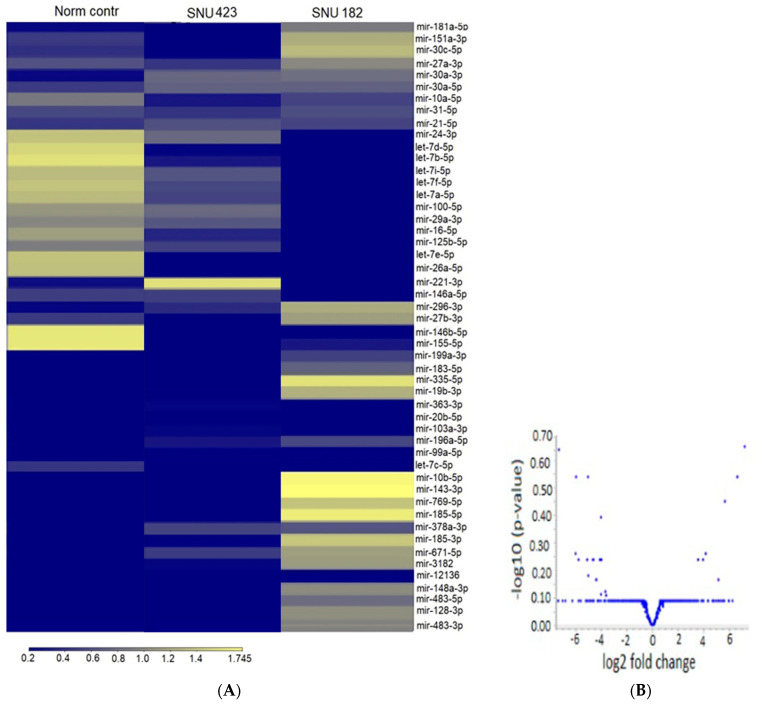
MicroRNA patterns of exosomes released from HBV-associated HCC cells. (**A**) The heatmap was constructed to identify exosomal miRNAs in HCC cells (SNU423 and SNU182, and normal liver cells THLE2). Columns display the clustering of exosome samples and rows indicate the clustering of miRNAs. The intensity of the color is proportional to the degree of up- or downregulation. The more similar the expression of the selected genes are between samples, the closer the samples are related in the dendrogram. (**B**) The volcano plot displays the log2 fold change of miRNA expression of the examined exosomes on the *x*-axis and –log10 *p*-value on the *y*-axis. Highly significant differences lie in the upper left and upper right-hand parts of the volcano plot high in the plot. Micro RNAs were considered significant with a fold change >1.5 and *p*-value < 0.05.

Next-generation sequencing (NGS) was applied to determine the miRNA expression of exosomes released by HBV-related HCC cell lines SNU423 and SNU182 in comparison with the normal liver cell line THLE2. Figure 2 shows the expression heatmap of the differentially expressed miRNAs in which the genes with similar expression patterns are grouped together and are connected by a series of branches (clustering tree or dendrogram), and the length of the branches reflects the degree of similarity [44]. The volcano plot in Figure 2 shows the relationship between the *p*-values of a statistical test and the magnitude of the difference in expression values of the samples in the groups. A total of 40 differentially expressed miRNAs (*p* value < 0.05) discriminate HBV-related HCC cells from the normal liver cells THLE2, of which 27 miRNAs are upregulated in comparison with the normal liver cells and 13 are downregulated. The top upregulated miRNAs in both HCC cell types included mir-483-5p, -145-5p, -143-3p, -3180, -185-3p, and -10b-5p with a Log fold change (FC) between 10, 14, and 7.53; and the top downregulated included mir-376a-3p, let-7e-5p, mir-195-5p, let-7d-3p, and mir-146b-3p with a logFC between −2.74 and −3.69 (Supplementary Table S1). The two HCC cell lines display distinctly different patterns of miRNA expression, despite common origins, and primary grade III–IV HCC tumors infected with hepatitis B virus-x [45,46]. For example, mir-483-3p and mir-143-5p are expressed only in SNU182, but not in SNU423 and THLE2, and mir-195-5p is not expressed in SNU-423, but was detected in SNU-182 and the normal lever cells.

**Figure 3:**
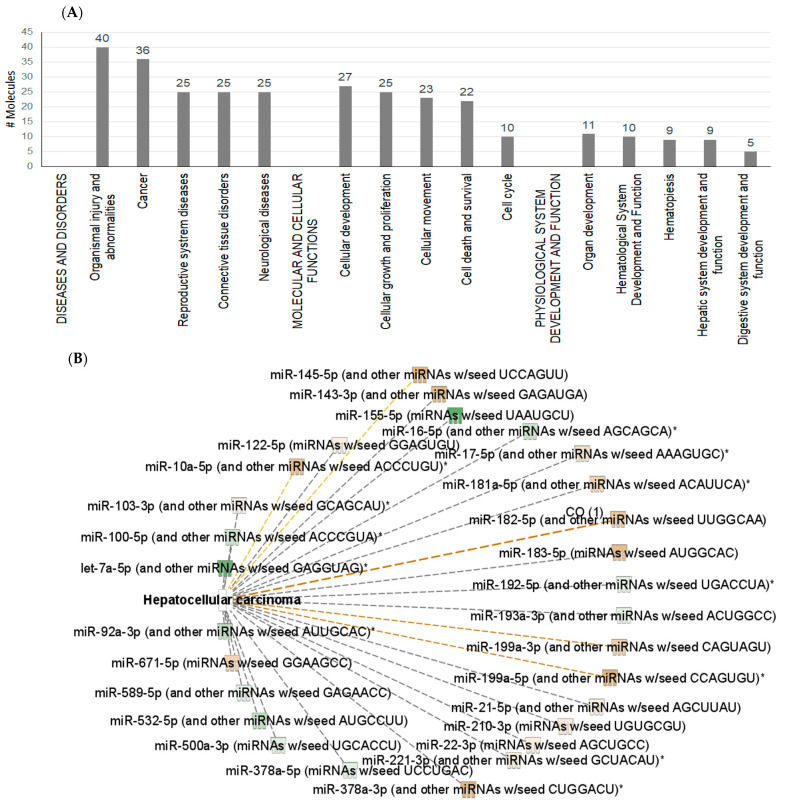
IPA identified impacts of the differentially expressed exosomal miRNAs on molecular mechanisms of diseases (**A**) and identified enrichment in several categories, related to organismal injury, cancer, cellular development, cell growth and proliferation, organ development, and hepatic system development and function. The image of (**B**) shows IPA prediction of correlation between differentially expressed miRNAs and hepatocellular carcinoma (HCC). Grey color indicates downregulation and yellow color indicates upregulation. Asterisks indicate that multiple identifiers in the dataset file map to a single gene in the IPA Global Molecular Network.

To better understand the role and functions of differentially expressed exosomal miRNAs and their predicted transcriptomic targets, we examined the possible downstream effects of miRNAs, based on published data using Ingenuity Pathway Analysis (IPA) and DIANA-mirPath softwares. DIANA-miPath is a web-based tool developed to identify miRNA-targeted pathway analysis [47]. IPA reports the biological mechanism in three aspects: “diseases and disorder”, “molecular and cellular functions”, and “physiological system development and function”. This analysis revealed enrichment in several categories related to organismal injury, cancer, cellular development, cell growth and proliferation, organ development, and hepatic system development and function (Figure 3A). Notably, in the category “cancer”, IPA predicted the activation of neoplasia, metastasis, and invasive cancer associated with the upregulation of miRNAs (i.e., mir-483, mir-378, mir-182, mir-16, mir-145, mir-10b) related to oncogenic functions, and specifically a correlation with HCC (Figure 3B).

**Figure 4:**
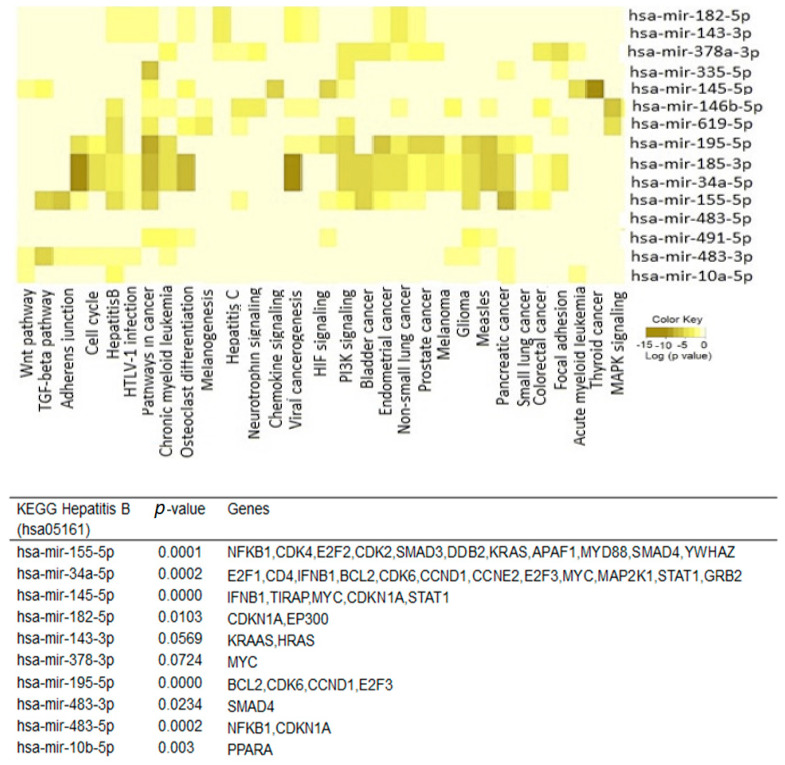
Heatmap showing the top 20 differentially expressed miRNAs versus KEGG (Kyoto Encyclopedia of Genes and Genomes) pathway. Heat map was created by DIANA-miRPath v3.0 miRNA target enrichment analysis. Grey colour represents lower enrichment *p*-values while shades of yellow represent higher enrichment *p*-values. The table displays the target genes of the top miRNAs.

DIANA mirPath v.3 software was used to align the top 20 miRNA-predicted targets with KEGG pathways. The KEGG (Kyoto Encyclopedia of Genes and Genomes) database is a collection of various pathways representing the molecular interactions and reaction networks. To identify the pathways involved, we mapped the KEGG database and found that the top 20 significantly dysregulated miRNAs were enriched in 30 pathways (Figure 4), that included enrichment of miRNA-predicted targets of hepatitis B (i.e., NFKB1, CDK4, E2F3, KRAS, MYC, KRAS, BCL2, CDKN1A, APAF1, MYD88, SMAD4, and others), hepatitis C (GSK3B, STAT3, NFKB1, PME3, EGFR, and others), viral carcinogenesis, as well as various cancers and signaling pathways related to cancer development.

**Figure 5:**
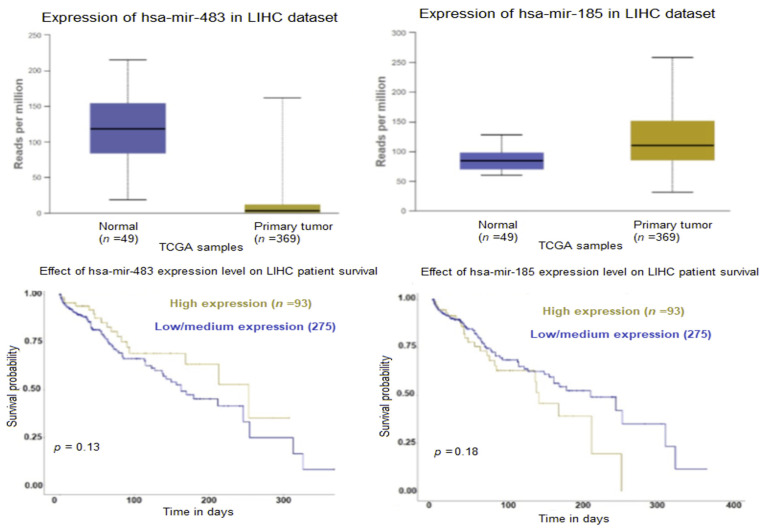
Expression profiles and patients’ survival analysis of the top upregulated miRNAs mir-483 and mir-185 in liver HCC tumors (UALCAN). Statistical significance of normal versus primary HCC tumor 1.322 × 10^−7^ (mir-483), <1 × 10^−12^ (mir-185). Kaplan–Meier plots (UALCAN) show the effect of miRNA expression in HCC on the overall survival of patients with primary HCC tumors.

We interrogated the TCGA (The Cancer Genome Atlas Program) miRNA expression database for the prognosis of several of the upregulated exosomal miRNAs in our dataset for HCC. TCGA data indicate that HCC tumors have lower expression of mir-483 and higher expression of mir-185 in comparison with normal liver tissue. Downregulation of both mir-483 and mir-185 conferred better survival probability, as shown in Figure 5.

### 2.3. Exosomal Proteins

**Figure 6:**
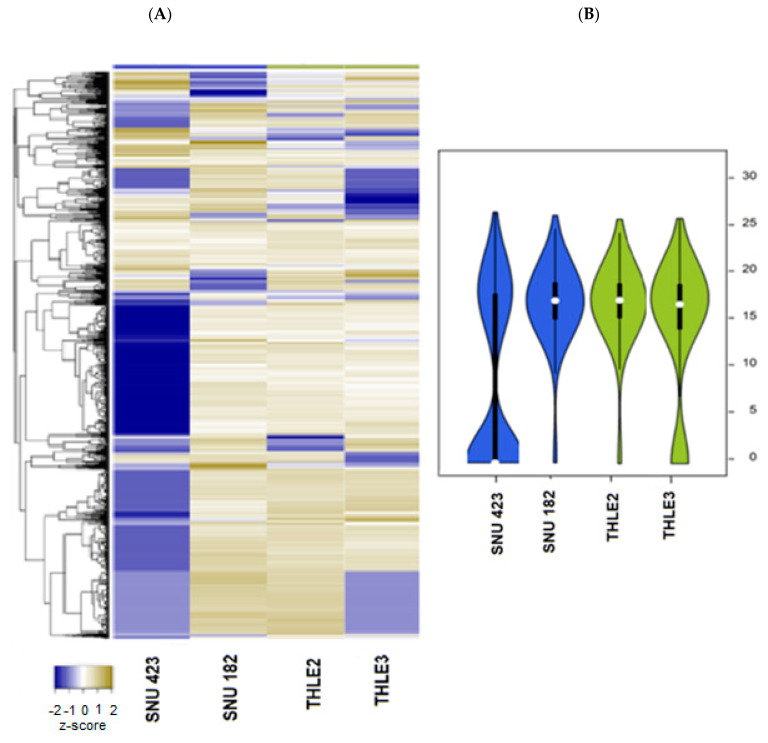
Proteome patterns of exosomes released from HBV-associated HCC cells. (**A**) The heatmap shows normalized exosomal protein intensities in HCC cell lines (SNU423 and SNU182) and normal liver cell lines (THLE2 and THLE3) (**B**). The intensity of the color is proportional to the degree of upregulation (yellow) and downregulation (blue)A violin plot of normalized.

The global protein profiling of the HBV-associated HCC cell lines SNU-423 and SNU-182 was examined using LC-MS/MS (liquid chromatography mass spectrometry). A total of 1730 proteins were identified to be expressed in the exosomes released by the HCC cells, of which 178 proteins were significantly different from the proteins released from the normal liver cells (FDR < 0.05) (Supplementary Table S2). The hierarchical clustering and violin plots representing the differentially expressed proteins, which discriminate HBV-HCC cells from normal liver cells (THLE2 and THLE3), are shown in Figure 6.

The top upregulated exosomal proteins in both HCC cell lines included STAM, PCOLCE, POSTN, EIF5, QDPR, IDH COL1A2, CORO1A, SNX9, F10, and EXOC8. More exosomal proteins were significantly downregulated in HCC cells compared to the normal liver cells. The top downregulated in both HCC cells included HPX, S100A6, IGH4, CAV1, IGL@, CD82, FN1, ITGA5, VAMP8.

**Figure 7:**
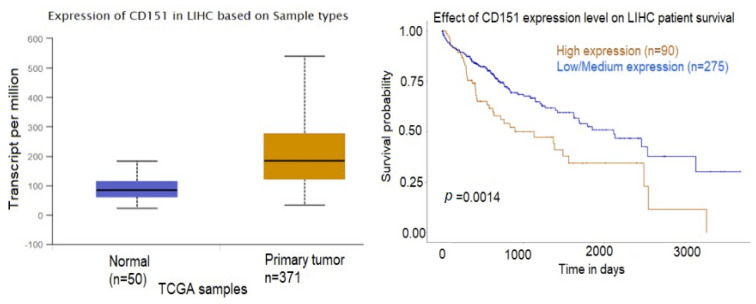
Expression profiles and prognostic value of CD151 in HCC. Statistical significance of normal tissue versus tumor 1.624 × 10^−12^. Kaplan–Meier plots (UALCAN) show the effect of protein expression on patients’ survival.

Commonly found “exosome-specific” markers included CD63, CD9, CD81, CD151 [48,49,50]. We could not detect CD63 in the exosomes released from the HCC cells, but it was found in the control liver cell lines; similarly, CD81 was not found in any of the examined exosomes (Supplementary Table S2). Another exosome classical marker, CD9, was found in all of the examined exosomes, and CD151 was found in the exosomes from both HCC cell lines and one of the normal liver cell lines (Supplementary Table S2). A search in the TCGA database showed that CD151 was highly expressed in HCC tumors compared to normal tissue and the higher expression was associated with worse survival (Figure 7). Actin, tubulin, and keratins have been observed in exosomes [51,52] and several of them, including ACTA1, TUBA1A+4A+1B+BB+, and KRT18, were also expressed in the exosomes from SNU-182, SNU-423, and normal liver cells in this study (Supplementary Table S2).

**Figure 8:**
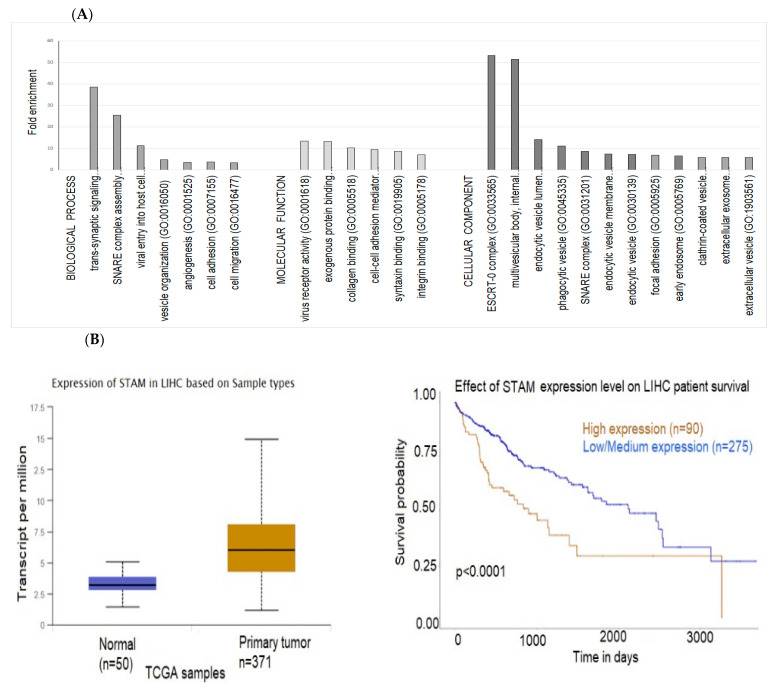
(**A**) Gene ontology (GO) enrichment analysis of the differentially expressed exosomal proteins. (**B**) Expression profiles and prognostic value of CD151 and STAM in HCC. Statistical significance of normal versus tumor 1.624 × 10^−12^ (CD151) and <1 × 10^−12^ (STAM). Kaplan–Meier plots (UALCAN) show the effect of gene expression in HCC tumors and normal liver tissue on patients’ survival.

Gene Ontology (GO) and Kyoto Encyclopedia of Genes and Genomes (KEGG) pathway enrichment analysis were performed to identify differentially expressed exosomal proteins using the DAVID (Database for Annotation, Visualization, and Integrated Discovery) online database. GO is a functional enrichment database used to search for enriched GO terms, which include molecular function (MF), biological process (BP), and cellular component (CC), while KEGG, which consists of a reference pathway database, is widely used for KEGG pathway mapping. The top enriched GO terms involved in biological process terms included regulation of renal phosphate excretion, trans-synaptic signaling, SNARE (soluble N-ethylmaleimide-sensitive fusion protein attachment protein receptors) complex assembly, and viral entry into host cell (Figure 8A). The most enriched molecular function terms were virus receptor activity, exogenous protein binding, collagen binding, and cell–cell adhesion activity; and the top enriched cellular component terms included the ESCRT-0 (endosomal sorting complex required for transport) complex, multivesicular body/internal vesicle, endocytic vesicle, and the SNARE complex (Figure 8A). STAM (signal-transducing adaptor molecule), a significantly overexpressed protein in both SNU-182 and SNU-423, is a component of both ESCRT-0 and SNARE and is involved in facilitating the first step in membrane invagination during EV formation [53]. A search in the TCGA database revealed that STAM is highly expressed in HCC tumors versus normal tissue and lower expression was associated with better survival in patients with HCC (Figure 8B).

**Figure 9:**
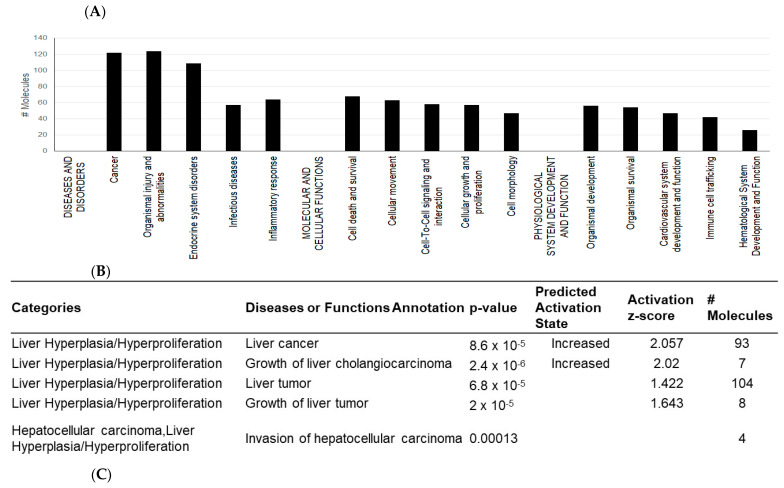

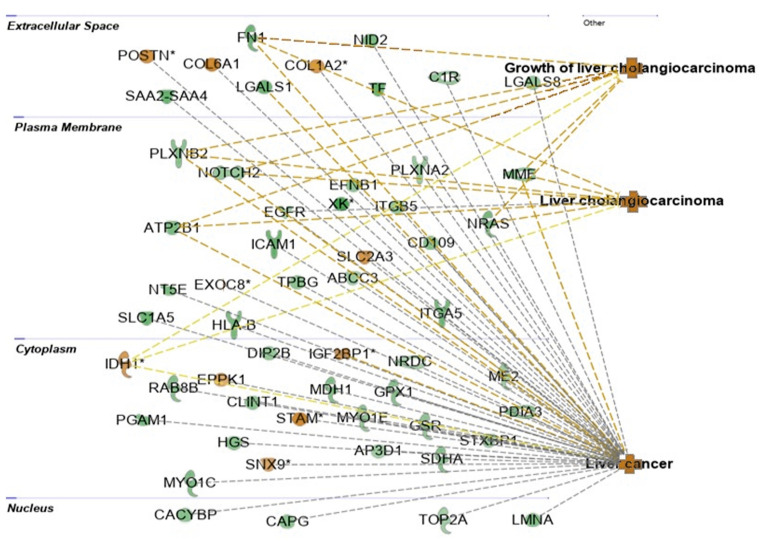
IPA functional analysis of the differentially expressed exosomal proteins (**A**). IPA predicted hepatotoxicity of differentially expressed proteins released from HVB-related HCC cells (**B**). IPA predicted a correlation between the expression of exosomal proteins and liver cancer, liver cholangiocarcinoma, and the growth of liver cholangiocarcinoma (**C**). Asterisks indicate that multiple identifiers in the dataset file map to a single gene in the IPA Global Molecular Network.

The differentially expressed exosomal proteins were further analyzed with the Ingenuity Pathway Analysis (IPA) database to identify diseases and disorders, molecular and cellular functions, and physiological system development and function. The results showed enrichment in cancer, organismal injury, cellular movement, cell-to-cell signaling, and organismal development. The most enriched diseases and disorders were cancer (122 molecules), organismal injury and abnormalities (124 molecules), and endocrine system disorders (109 molecules). Based on the expression of the exosomal proteins, IPA predicted an increased probability of digestive system cancer (z-score = 2.061). The most represented molecular and cellular functions were cell death and survival (68 molecules), cellular movement (63 molecules), and cell-to-cell signaling and interaction (58 molecules). In category physiological system development and functions, the most important were organismal development (56 molecules), organismal survival (54 molecule), and cardiovascular system development and function (47 molecules). The most significant are presented in Figure 9A. In the category “Tox functions”, IPA predicted increased liver hyperplasia/hyperproliferation and increased liver cholangiocarcinoma (Figure 9B). The correlation between the exosomal protein expression and liver cancer and liver cholangiocarcinoma is presented in Figure 9C.

### 2.4. Interactions between Exosomal miRNAs and Proteins

Integrated miRNA–mRNA–protein analyses have been the focus in many studies on cancer [54,55,56]. miRNAs have both direct and indirect effects on protein expression at any stage of a signaling pathway and may include the involvement of miRNAs in feedback loops and feedforward cascades with transcription factors and signaling molecules [57]. We have analyzed the interactions between the exosome differentially expressed miRNAs (*p*-value < 0.05) and exosomal proteins (*p*-value < 0.05) using a hypergeometric test [58] of the online software miRNet 2.0 to determine the miRNA–protein co-expression networks. miRNet identified the important functional modules and the higher ranked miRNAs/proteins in the network based on Degree centrality, Closeness centrality, Betweenness centrality, and Stress centrality [59]. Degree centrality assigns an importance score based simply on the number of links held by each node. Degree tells us that how many direct, “one hop” connections each node has to other nodes in the network. It is the simplest measure of node connectivity. Sometimes, it is useful to look at in-degree (number of inbound links) and out-degree (number of outbound links) as distinct measures, e.g., when looking at transactional data or account activity. Closeness centrality scores each node based on their “closeness” to all other nodes in the network. Closeness measure calculates the shortest paths between all nodes and then assigns each node a score based on its sum of shortest paths. It is used for finding the factors that are best placed to influence the entire network most quickly. Betweenness centrality measures the number of times a node lies on the shortest path between other nodes. Betweenness tells us which nodes are “bridges” between nodes in a network. It does this by identifying all the shortest paths and then counting how many times each node falls on one. It is useful for analyzing communication dynamics. The stress of a node in a biological network, for instance, a protein-signaling network, can indicate the relevance of a protein as functionally capable of holding together communicating nodes. The higher the value is, the higher the relevance of the protein in connecting regulatory molecules.

**Figure 10:**
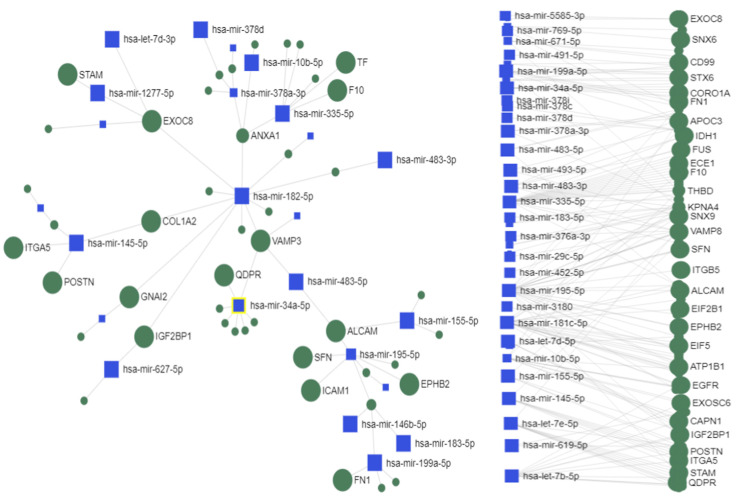

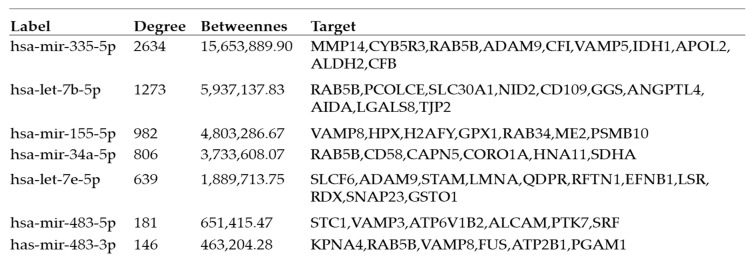
Exosome miRNA–protein interaction Minimum/Steiner Forest Network (miRNet). The table shows the central miRNAs and their targets. The blue squres represent the selected 30 dysregulated miRNAs and the graycircles represent the selected 30 exosome dysregulated proteins.

miRNet identified a total of 231 nodes. The miRNA–protein network analysis resulted in seven miRNAs (has-mir-335-5p, has-let-7b-5, has-mir-155-5p, has-mir-34a-5p, has-let-7e-5p, hsa-mir-483-5p, has-mir-483-3p) with centrality and with Degree >145. The Minimum Network and Steiner Forest Network are presented on Figure 10. Both the Minimum Network and Steiner Forest Network tools aim to construct a minimally connected network that contains all of the seed genes. This means that the only added nodes are ones that connect previously disjointed networks of seed genes. The difference between the Minimum Network and the Steiner Forest Network is the way in which the approximate solution is computed. For the minimum network, miRNet implements an approximate approach based on shortest paths: the software computes pair-wise shortest paths between all seed nodes, and removes the nodes that are not on the shortest paths. For the Steiner Forest Network, miRNet implements a fast heuristic prize-collecting Steiner Forest algorithm.

The KEGG and REACTOME enrichment analysis revealed an association with focal adhesion, extracellular matrix (ECM) receptor interaction, axon guidance, vesicle-mediated transport, and VEGF signaling (Table 1), indicating the important role of HBV-HCC-released exosomes in the promotion of invasion and metastasis cascade including angiogenesis, EMT, invasion, migration, and establishment of a premetastatic niche. One of the central nodes is the activated leukocyte cell adhesion molecule ALCAM (CD66), a target of multiple of the dysregulated miRNAs, including mir-18, mir-34, mir-144, mir-135 (Supplementary Table S1). ALCAM is not expressed in any of SNU-182 and SNU-423 cell lines, but is expressed in the normal liver cells (Supplementary Table S2).

## 3. Discussion

In this study, we have used NGS and LC–MS)/MS to assess the global signature of miRNAs and proteins in exosomes released from HBV-related HCC high-grade human cell lines SNU423 and SNU182. We have identified distinct miRNA and protein signatures, which distinguished SNU423 and SNU182 from normal liver cell lines (THLE2 and THLE3).

The analysis of the dysregulated exosomal miRNAs showed that the top dysregulated miRNA mir-483-5p was upregulated in both examined cell lines SNU-423 and SNU182. Overexpression of mir-483 has been detected in advanced cirrhosis patients infected with hepatitis C virus [60] and patients with HCC [61]. The integrated miRNA-protein analysis showed that ALCAM (activated leukocyte cell adhesion molecule), a target of mir-483-5p, is one of the central nodes in the co-expression network, which is not expressed in the exosomes of the examined SNU423 and SNU182 cells in this study. ALCAM (known also as CD166) is a cell surface adhesion glycoprotein related to immunoglobulins that modulates cell–cell interactions and is found at sites of cell–cell junction, with an important role in cancer progression and metastasis [62,63,64]. High expression of ALCAM/CD166 was associated with progression, metastasis, and response to therapy in several cancers [65], but lower expression in other cancers, such as breast cancer correlated with an aggressive phenotype and worse prognosis [66,67]. Recent study by Li et al. [68] showed that the high expression of mir-483-5p in HCC tumors was associated with downregulation of ALCAM. These findings along with our data showing no presence of ALCAM in exosomes released by SNU-423 and SNU182 suggest that the exosomal mir-483-5p/ALCAM axis may be an important regulator in invasion and metastasis of HVB-related HCC.

Our literature search showed that commonly dysregulated miRNAs in HBV-associated HCC include upregulated mir-18a, mir-21, mir-221, and mir-222 and downregulated mir-26a, mir-125, mir-122 [8,69,70,71,72], which combined target-to-target pathways involved in the increased proliferation and reduced apoptosis in HCC, such as WNT/beta-catenin, PI3K/Akt, MAPK, TP53, and JAK/STAT [73]. Hepatocytes infected with HBV produced exosomes containing miR-21, miR-192, miR-215, miR-221, and miR-222, and inhibited T cells’ secretion of IL-21, an important inflammatory molecule of hepatitis immunity [33]. Several of these miRNAs known to induce dysregulation were found in the exosomes released by the HCC cell lines in this study including upregulation of mir-221-3p, mir-21-3p, mir-222-5p, and mir-18a-3p. Overexpression of mir-1269 and its correlation with poor prognosis has also been reported in various cancers, including HCC [74]. mir-1269b has been associated with HBx-induced promotion of proliferation and migration of HCC in an NF-kb-dependent manner [75] and replication of HBV through interaction with c-Myc [76].

The proteins released by exosomes can change the gene expression and functions of recipient cells, potentially driving the process tumor formation, proliferation, and metastasis [77]. Using a proteomic approach, we identified a number of highly expressed exosomal proteins derived from HBV-related HCC cells. However, of the common exosome markers (CD63/CD9/CD81/CD151), we only detected them in the HCC cells CD151 and CD9 in the HCC. This finding may be related to the evidence in support of the heterogeneity of the small EVs and the presence of a subpopulation of CD63/CD81/CD9-negative exosomes [78,79,80]. Moreover, CD63 knockdown induced a significant increase in exosome production, thus confirming CD63 as an important part of multivesicular endosome (MVE) biogenesis and/or trafficking [81]. However, CD151 has been shown to regulate integrin adhesion activity and extracellular matrix (ECM) remodeling [82] and to be involved in neoangiogenesis and cancer metastasis [83,84], including HCC [85,86].

The analysis of the exosomal proteins showed enrichment in terms associated with viral receptor activity and invasion, vesicle formation and adhesion, cell–cell adhesion, and ESCRT and SNARE complexes, which are consistent with the role of exosomes in viral infections [87,88,89] and in malignant transformations of normal cells [90,91,92,93]. ESCRT machinery plays a prominent role in exosome biogenesis and SNARE proteins are important in exosome secretion [94,95,96]. Enveloped viruses recruit ESCRT machinery through the function of specific peptide motifs within their structural proteins [97,98,99,100]. HBV is an enveloped, DNA-containing pararetrovirus that requires ESCRT to exit cells [101,102,103]. Upon infection of liver cells, the partially double-stranded 3.2 kb DNA genome is converted to the covalently closed circular DNA inside the nucleus and serves as a template for the transcription of the pregenomic (pg) RNA and three subgenomic RNAs that are exported to the cytoplasm [104]. Several SNARE proteins were differentially expressed in the exosomes released from the HBV-related HCC cell lines in this study, including STAM, EXOC8, SNX9, CORO1A, VAMP8, VAMP3, STX6, MMP12, EPHB2, and APOL2. Positive correlation between the expression of POSTN [105,106,107,108], COL6A1, [109,110,111], EXOC8 [112,113], and STAM [112] and negative correlation with NOTCH2 [114] have been associated with tumor size, grade, and lymph node metastasis in other cancers. POSTN, which functions as a ligand for aV/b3 and aV/b5 integrins, is involved in the adhesion and migration of multiple cell types associated with angiogenesis and metastasis [115,116]. The SNARE-binding proteins VAPA and VAPB, which were expressed in the exosomes released from SNU-182 cells, have been shown to enhance the replication of hepatitis C virus (HCV) through interaction with the viral proteins NS5A and NS5B [87,117].

The integrated enrichment analysis showed that most of the differentially expressed exosomal miRNAs and proteins were significantly enriched in the promotion of invasion and metastasis cascade, such as focal adhesion, vesicle-mediated transport, ECM receptor interaction, axon guidance, angiogenesis, and establishment of a premetastatic niche. We have previously demonstrated that HCC cells released exosome-induced differentiation and angiogenic activity of endothelial cells [30]. Among the central protein nodes in the co-expression network is EPHB2, a member of the Ephin receptor family of receptor tyrosine kinase transmembrane glycoproteins, involved in angiogenesis [118]. EPHB2 can function as both tumor promoter and suppressor in different cellular contexts and is downregulated in our dataset, a finding that correlates with reports showing that inactivation of EPHB2 promoted cell proliferation and invasion in certain types of cancers [119]. For example, a study on gastric cancer (GC) showed that as the tumor grade increased, the expression rates of EPHB2 lowered significantly, and the loss of EPHB2 expression was significantly associated with poor survival of GC patients [120].

Collagens, a major component of the ECM, have been involved in carcinogenesis in various tissue types, mostly predictive of poor prognosis [121]. The high expression of COL1A2 and low expression of let-7g have been found in HCC clinical specimens and correlated with poor prognosis [117]. The integrated analysis of exosomal miRNAs and proteins in this study suggested a correlation between the upregulated mir-199a and mir-145, and their target COL6A1. Upregulation of COL6A1, which is an extracellular matrix protein, has been reported to enhance motility and metastasis in some cancers [122], but there is no data on its effect in HVB-related HCC.

The significantly expressed miRNAs and proteins in exosomes released from the HCC cells in this study were distinctly different between the two cell line studies. Both cell lines originated from primary high-grade HCC (grade III–IV) tumors from adult male Asian patients, infected with hepatitis B virus-x [45,46]. Both cell lines have mutations in the *p53* gene (SNU-423 intron5/exon5 junction deletion 126~132, mutation AG to GG; SNU-182 codon 215 AGT to ATT) [45]. However, they exhibited a clearly different pattern of exosomal miRNA and protein expression. Genomic heterogeneity of tumor cells with similar origin has been linked to genetic instability followed by subclonal evolution, epigenetic plasticity, diverse microenvironmental factors, and heterotypical interactions with immune and stromal cells [123]. Several studies have suggested that distinct genetic and molecular subtypes can often co-exist within the same tumor [124,125], which are associated with differences in progression and metastasis, as well as in the response to therapy [123]. In addition, during prolonged cell culture, many cells diverge from their original phenotype (transdifferentiation) [126]. An analysis of the mutational profiles of SNU423 and SNU182 showed mutations at chromosomes 3, 13, and 19 in SNU182 and unrelated mutations at chromosomes 3 and 13 of SNU423 [127]. Another study on the expression of vimentin and e-cadherin, markers of epithelial-mesenchymal transition (EMT), showed expression of e-cadherin in SNU423, but not in SNU182, suggesting that the EMT status of SNU423 may be classified as epithelial, while SNU182 is a mesenchymal line [128].

In this study, we have compared the miRNA and protein expression in exosomes released from two HBV-related high-grade cell lines, SNU423 and SNU182, derived originally from the same type of tumor. Importantly, we have identified specific patterns of miRNA and protein expression in exosomes released from SNU-423 and SNU-182 in comparison with two normal liver cell lines, some of which are in agreement with the published data indicating that HCC is a very heterogeneous disease, reflecting multiple etiologies [129]. The variations in the expression pattern between the HCC cell lines might help in their potential application as model systems for tumor types and specifically in the studies on sensitivity to target therapies.

## 4. Materials and Methods

### 4.1. Cell Lines and Culture

Human HCC cell lines SNU-423 (ATCC-CRL-2238) and SNU-182 (ATCC-CRL-2235), and human immortalized liver cell lines THLE-2 and THLE-3, were obtained from the American Type Cell Collection (ATCC, Manassas, VA, USA). HCC cells were grown in RPMI culture media (Lonza, Walkersville, MD, USA), supplemented with 10% heat-inactivated fetal bovine serum (FBS) (Life Technologies, Waltham, MA, USA). THLE2 and THLE3 cells were cultured in a BEGM Bullet kit (Lonza), which contains BEBM basal medium and supplements. The final growth medium consists of BEBM supplemented with 10% FCS, bovine pituitary gland extract, hydrocortisone, epidermal growth factor (EGF), insulin, triiodothyronine, transferrin, and retinoic acid. THLE cells require a special coating medium that consists of the following: RPMI1640 without glutamine supplemented with 0.01 mg mL^−1^ bovine serum albumin, (heat shock fraction, Sigma, Burlington, MA, USA), 0.03 mg mL^−1^ type I collagen from bovine skin (Sigma), and 0.01 mg mL^−1^ fibronectin from human plasma (Sigma). All cell lines were maintained independently in the recommended medium at 37 °C and 5% CO_2_.

### 4.2. Exosome Purification

Exosomes were isolated from culture media, collected 48 h after cell starvation using sequential centrifugation or by ExoQuick-TC-ULTRA (System Biosciences, Palo Alto, CA, USA). The ultracentrifugation was performed as previously described [30] and included centrifugation at 3000× *g* for 15 min, followed by 10,000× *g* for 30 min and 110,000 for 3 h. The resulting exosome pellet was washed in phosphate-buffered saline (PBS) and centrifuged again at 110,000× *g* for 70 min. The final pellet was resuspended in PBS and used in further experiments. Exosome isolation by ExoQuick -TC ULTRA was performed following manufacturer’s instruction and included removal of cell debris at 3000× *g* for 15 min and precipitation with the ExoQuick-TC reagent overnight at 4 °C. After incubation, the solution was centrifuged at 3000× *g* for 10 min and exosomes were added to ExoQuick ULTRA columns, which were centrifuged at 1000× *g* for 30 s. The exosomes were then collected and diluted in PBS. The protein concentration of exosomes was determined by a Bradford assay (BioRad, Hercules, CA, USA).

### 4.3. Nanoparticle Tracking Analysis (NTA)

Hydrodynamic diameter (dH) and particle concentration were determined using a Zetasizer 3000 (Particle Metrix GmbH, Ammersee, Germany). Exosomes were diluted in 0.2 µm filtered PBS at 500× dilution to obtain ~100 particles per field of view using a sensitivity of 85, frame rate of 30, and shutter of 100 and a 488 nm laser source. The measurements were taken in 11 unique locations throughout the viewing window and compiled by the software. Concentration weighted distributions were used to determine median and mean hydrodynamic diameter.

### 4.4. Exosome microRNA Analysis

#### 4.4.1. MicroRNA Isolation

Total RNA, including miRNA, was isolated from 1 mL cell culture media samples using exoRNeasy Midi kit (QIAGEN) following manufacturer’s instructions. Briefly, pre-filtered cell culture medium (0.8 μm syringe filter) was mixed 1:1 with 2× binding buffer (XBP) and added to the exoEasy membrane affinity column to bind the EVs to the membrane. After centrifugation, the flow-through was discarded and a wash buffer (XWP) was added to the column to wash off non-specifically retained material. After another centrifugation and discarding of the flow-through, the vesicles were lysed by adding QIAzol to the spin column, and the lysate was collected by centrifugation. Following the addition of chloroform and thorough mixing and centrifugation to separate organic and aqueous phases, the aqueous phase was recovered and mixed with ethanol. The sample ethanol mixture was added to a RNeasy MinElute spin column and centrifuged. The column was washed once with buffer RWT, and then twice with buffer RPE, followed by elution of RNA with water. The purity and concentrations of total RNA of the plasma samples were measured with a NanoDrop ND-1000 spectrophotometer. The RNA yield and size distribution were analyzed using an Agilent 2100 Bioanalyzer with an RNA 6000 Pico kit (Agilent Technologies, Foster City, CA, USA).

#### 4.4.2. Next Generation Sequencing (NGS)

NGS libraries were constructed using a QIAseq miRNA library. Briefly, 3’ and 5’ adapters were ligated to mature miRNAs. The ligated miRNAs were then reverse transcribed to cDNA using a reverse transcription (RT) primer with unique molecular indices (UMI). After library amplification, a cleanup of the miRNA library was performed using a streamlined magnetic bead-based method and quality control (QC). The library was than sequenced on Illumina NextSeq 500/550 equipment.

#### 4.4.3. Data Analysis

Sequence data were converted to FASTQ files, analyzed using CLC Genomics Workbench (v.12.02), and UMIs were extracted. Reads were mapped to MiRNA database miRbase v22 and human genome GRCh38 version 97. Differential expression analysis was preformed via the Bioconductor Package DESeq2, including hierarchical clustering plus a heatmap, principal component analysis, normalization based on median ratios of mean miRNA expression, and the Benjamin–Hochberg method to correct for false discovery rate (FDR). miRNAs with FDR < 0.05 and log fold change (FC) > 1.0 were considered significant. miRNA transcriptome targets were identified by the miRNet (https://www.mirnet.ca/, accessed on 21 June 2023) and TargetScan (http://www.targetscan.org/vert_71/ accessed on 10 January 2023 and 15 April 2023) online analysis tools, which rely on the identification of the seed region between the miRNA and the corresponding target genes [130].

### 4.5. Exosome Proteomic Analysis

#### 4.5.1. Liquid Chromatography with Tandem Mass Spectrometry (LC–MS)/MS)

To characterize the proteomic profiles of the exosomes released by HCC cells and control liver cells, we conducted LC-MS/MS. Tandem Mass Tag (TMT) 10-plex reagent (ThermoFisher, Waltham, MA, USA) was used for exosome proteomics analysis. Briefly, protein samples were reduced, alkylated, and purified by chloroform/methanol extraction prior to digestion with sequencing-grade modified porcine trypsin (Promega, Maddison, WI, USA). Tryptic peptides were then separated by reverse phase XSelect CSH C18 2.5 µm resin (Waters, Milford, MA, USA) on an in-line 150 × 0.075 mm column using an UltiMate 3000 RSLCnano system (Thermo). Peptides were eluted using a 90 min gradient from 98:2 to 65:35 buffer A:B ratio (Buffer A = 0.1% formic acid, 0.5% acetonitrile; Buffer = 0.1% formic acid, 99.9% acetonitrile). Eluted peptides were ionized by electrospray (2.4 kV) followed by mass spectrometric analysis on an Orbitrap Eclipse Tribrid mass spectrometer (Thermo). MS data were acquired using the FTMS analyzer in profile mode at a resolution of 120,000 over a range of 375 to 1400 *m*/*z* with advanced peak determination. Following HCD activation, MS/MS data were acquired using the ion trap analyzer in centroid mode and normal mass range with a normalized collision energy of 30%.

#### 4.5.2. Data Analysis

Proteins were identified by database search against the UniprotKB database restricted to *Homo sapiens* (September 2020) using MaxQuant (version 1.6.17.0, Max Planck Institute). The database search parameters included selecting the MS1 reporter type, trypsin digestion with up to two missed cleavages, fixed modifications for carbamidomethyl of cysteine, variable modifications for oxidation on methionine and acetyl on N-terminus, the precursor ion tolerance of 5 ppm for the first search and 3 ppm for the main search, and label-free quantitation with iBAQ. Scaffold Q+S v.5.3.0 (Proteome Software) was used to verify MS/MS-based peptide and protein identifications. Protein identifications were accepted if they could be established with less than 1.0% false discovery and contained at least 2 identified peptides. Protein probabilities were assigned by the Protein Prophet algorithm [131]. MaxQuant iBAQ intensities for each sample were assessed for quality and differential abundance using proteoDA [132,133]. The data were normalized using cyclic loess [134] and statistical analysis was performed using linear models for microarray data (limma) with empirical Bayes (eBayes) smoothing to the standard errors [134]. Proteins with an FDR adjusted *p*-value < 0.05 and a fold change >2 were considered significant.

### 4.6. Functional and Pathway Analysis of Differentially Expressed Exosomal miRNAs and Proteins

The functional characterization of the differentially expressed miRNAs and proteins was performed using Kyoto Encyclopedia of Genes and Genomes (KEGG) pathway (http://www.genome.jp/kegg/pathway.html, accessed on 5 June 2023) and gene ontology (GO) enrichment evaluation based upon the Database for Annotation, Visualization, and Integrated Discovery (DAVID) (https://david.ncifcrf.gov/, accessed on 15 July 2023). GO enrichment analysis of annotated proteins and miRNA targets included the categories of Cellular Components (CC), Molecular Functions (MF), and Biologic Processes (BP). The pathway enrichment analysis was performed using Ingenuity Pathway Analysis (IPA) and a protein–protein interaction (PPI) network was generated using the String database web tool. miRNA functional analysis and the interaction between exosome miRNAs and exosome expressed proteins was analyzed using DIANA-miRPath v.3 [47] and miRNet 2.0 software [59].

## 5. Conclusions

Taken together, the results from this study suggest that exosomes released from HBV-related HCC high-grade cells have unique miRNA and protein expression profiles compared to normal liver cells. These exosomes appear to be CD9-positive, CD63/CD81-negative exosomes, enriched with oncogenic factors such as COL1A2, POSTN, STAM, COL6A1, EXOC8, mir-483, and other oncomiRs. Both HCC cell lines, despite the same origin of tumor tissue with similar pathological characteristics, showed quite different patterns of exosomal miRNA and protein expression, which might be associated with the different etiology of HCC. While these results are only a small sample of two malignant and two normal liver cell lines, the data provide a basis for future in-depth studies on HBV-HCC tumor-derived exosomes, including clinical samples and statistically powered sample sizes, which will aid our understanding of the role of HBV infection in the development of HCC, and in the development of new targets for treatment or non-invasive predictive biomarkers.

## Figures and Tables

**Table 1:** KEGG and REACTOME exosome miRNA–proteins co-expressed signaling pathways.

Pathways	Molecules
ECM-receptor interaction and focal adhesion	COL1A2, COL6A1, FN1, ITGA5, ITGB3, ITGB1
Cell adhesion and Axon guidance	ALCAM, EPHB2, ITGA5, ITGB3, NRAS
Vesicle-mediated transport	EXOC8, COL1A2, SFN, STAM
Cell surface interactions at the vascular wall	ATP1B3, COL1A2, FN1, ITGA5, ITGB3, NRAS
P53 signaling pathway	CD82, SFN
SNARE interactions in vesicular transport	VAMP3
VEGF signaling	NRAS
MAPK signaling	NRAS, SRF

## Data Availability

Not applicable.

## Supplementary Materials

The following supporting information can be downloaded at: https://www.mdpi.com/article/10.3390/ijms241713098/s1.

## Abbreviations

HCC, hepatocellular carcinoma; HBV, hepatitis B virus; pgRNA, pregenomic RNA; NTA, nanoparticle analysis; NGS, next-generation sequencing; miRNA, microRNA; LC-MS/MS, liquid chromatography mass spectrometry; KEGG, Kyoto Encyclopedia of Genes and Genomes; TCGA, The Cancer Genome Atlas Program; GO, gene ontology; DAVID, Database for Annotation, Visualization, and Integrated Discovery; SNARE, soluble N-ethylmaleimide-sensitive factor attachment protein receptor; ESCRT, endosomal sorting complex required for transport ECM, extracellular matrix
